# Relative Age Effect Among the Best Norwegian Track and Field Athletes of All Time: Comparisons of Explosive and Endurance Events

**DOI:** 10.3389/fpsyg.2022.858095

**Published:** 2022-07-12

**Authors:** Alexander Kirkeberg, Truls Valland Roaas, Hilde Gundersen, Terje Dalen

**Affiliations:** ^1^Department of Physical Education and Sport Science, Faculty of Teacher Education and Arts, Nord University, Levanger, Norway; ^2^Department of Sport, Food and Natural Sciences, Western Norway University of Applied Sciences, Campus Bergen, Bergen, Norway

**Keywords:** track and field, RAE, sport-performance, athletics-development, physical factors

## Abstract

The purpose of this study was to investigate the degree of relative age effect (RAE) among the best Norwegian track and field athletes of all time, aged 13 years to senior, as well as to investigate the differences between athletes in events that impose different demands on their physical characteristics, categorised in endurance and explosiveness. The degree of RAE was investigated by examining the difference between the sample’s (*N* = 21,711) quarterly birth distribution and the quarterly distribution of birth of the Norwegian population as a whole from 1966 to 2019. To determine whether or not an RAE was present, chi-square tests (χ^2^) were conducted against an even distribution, with Cramer’s V (phi or *ɸ*) as a measure of effect size. The study’s results show a strong RAE in the two youngest age groups in both genders. RAE decreases with increasing age, but the effect is still present at the senior level in both men and women. Furthermore, the degree of RAE was strongest in explosive events in both boys (*ɸ* = 0.46) and girls (*ɸ* = 0.30), while in endurance events it was strong in boys (*ɸ* = 0.38) but not in girls (*ɸ* = 0.13). Prominent effect of RAE in the 13- and 14-year-old classes can be explained by the fact that in the youngest age groups impose the highest relative age difference. In addition, this is an age group where there are large differences in growth spurts, physical characteristics and training experience. Elimination of RAE with increasing age may be due to the fact that after puberty inherent physical advantages as a result of the month of birth are evened out. The prominent RAE in explosive events and in boys may be due to the fact that puberty and growth spurts make boys faster, stronger and larger, while puberty and growth spurts in girls are not always beneficial for girls in track and field events. The practical significance of the results relates to athletes developmental opportunities. Irrespective of whether young track and field athletes are relatively older or younger they should be met with patience and dedication from coaches. Superficial short-term categorization of young athletes potential do more harm than good.

## Introduction

In Norwegian track and field, it is common to divide youth athletes into chronological age groups, which in practice means that 13-, 14-, 15-, 16- and 17-year-olds compete in their own chronological age classes (NFIF, 2020a^1^; NFIF, 2021a^2^). The reason for having such a class division in accordance with increasing age is the desire to erase the differences in maturation between the athletes (Helsen et al., 2005; Cobley et al., 2009). During adolescence, physical characteristics such as speed, resilience, strength and endurance will change as a result of natural maturation processes (Malina et al., 2010; Handelsman, 2017). Thus, having athletes of the same age compete against each other is an attempt to reduce some of the maturation differences so the athletes can compete against each other on a fairer and more equal basis. Although such class divisions will contribute to fairer competition between the athletes, there will still be differences in the development of physical and mental characteristics between those athletes competing in the same class. The effects of the advantages and disadvantages of being relatively older in sports are described as the relative age effect (RAE; Musch and Grondin, 2001). Several studies show that being relatively older, i.e., being born earlier in the year, will have a positive impact on sports performance (Malina et al., 2004b, 2007; Gil et al., 2014). Nonetheless, studies of RAE in sports also show that being relatively older can be a disadvantage, especially among older female adolescents in weight-bearing sports like gymnastics (Hancock et al., 2015).

In track and field, relative age advantages and disadvantages may be highly apparent, as performance in the various events is determined by the direct measurement of skills relating to, for example, explosiveness or endurance. In many sports where height, body mass, strength and power are an advantage, that is, explosive sports, the early maturing boy or girl at any given age is likely to have a biological advantage over those who mature later (Baxter-Jones, 1995). The individual’s maturation together with external facilitating procedures (i.e., selection) in sports are suggested to influence the individual’s ability to invest time in practice and to accumulate sport-specific skills and experience, factors which are critical for long-term performance (Baker and Horton, 2004). The available research on RAE in explosive track and field events indicate a strong RAE in both boys and girls in the youngest classes among high-level athletes (Hollings et al., 2014; Romann and Cobley, 2015; Redondo et al., 2019). Furthermore, previous RAE research on explosive events in track and field shows that RAE is stronger in boys than in girls (Kearney et al., 2018; Brustio et al., 2019; Redondo et al., 2019; Figueiredo et al., 2021). For example, a study conducted by Redondo et al. (2019) shows that RAE among 13-year-old Spanish throwing athletes in track and field is doubled in boys (*ɸ* = 0.8) compared to girls (*ɸ* = 0.4). In another study, Kearney et al. (2018) documented similar evidence of RAE in older adolescents in sprints and hurdles, when they—by using odds ratio (OR) comparisons—found twice as big an OR in boys U17 (OR K1:K4 = 3.0) than in girls U17 (OR K1:K4 = 1.5). Although the majority of the studies on RAE in explosive disciplines in track and field constitue a high degree of RAE among both boys and girls, Brustio et al. (2019) found no clear evidence of RAE in the pole vault among older female adolescents, which can be due to the fact that puberty and increased fat mass decrease relative strength among females (Hewett et al., 2004).

Studies investigating the prevalence of RAE in endurance track and field events show that RAE is weaker in endurance events than in explosive events. For example, the studies conducted by Kearney et al. (2018), Brustio et al. (2019) and Figueiredo et al. (2021), which analysed the degree of RAE in both explosive and endurance events across both genders, indicate that RAEs in the endurance events are considerably weaker, especially among girls. Kearney et al. (2018) documented that U13 boys were 14.6 times more likely to be in the top 20 in British athletics in sprint and hurdle disciplines if they were born in Q1 of the year and 4.0 times more likely to be in the top 20 in middle-distance events if they were born in Q1 than in Q4. In the same study, Kearney et al. (2018) found that U13 girls were 6 times more likely to be in the top 20 in sprint and hurdle events if they were born in the first quarter of the year instead of the last but only 3.6 times more likely to be in the top 20 in middle-distance events if they were born in Q1 than in Q4. Similar findings are documented in older adolescents, for example, by Figueiredo et al. (2021) who analysed the degree of RAE among Brazilian U20 athletes and found a medium RAE in sprints and hurdles (*ɸ* = 0.28) and a small RAE in long-distance (*ɸ* = 0.17) events for boys, while among girls, only a small RAE was found in sprints and hurdles (*ɸ* = 0.18) and no RAE at all was found in long-distance (*ɸ* = 0.04) events. No RAE in the long-distance disciplines among older female adolescents can be due to the fact that increased fat mass has a negative impact on their relative endurance, which can weaken both their mental (Robinson and Ferraro, 2004) and physical (Szczesny, 1993; Rogol et al., 2000) performance in long-distance events and other sports where relative endurance is important.

Previous studies have indicated that these differences in the degree of RAE between gender and physical characteristics can be explained by mechanisms such as puberty (Malina et al., 2010), competition (Musch and Grondin, 2001) and psychological maturation (Hancock et al., 2013). For example, a greater degree of RAE among boys in explosive track and field events can be explained by boys experiencing a significant increase in testosterone levels (Beunen and Malina, 2007; Handelsman, 2017). Higher testosterone levels are beneficial in explosive track and field events because they make boys stronger, faster and taller, which would give a large advantage in most of the explosive disciplines in track and field (Papaiakovou et al., 2009; Carvalho et al., 2017). Boys will have 10–15 times more testosterone than girls after puberty (Handelsman et al., 2016), which can explain why boys have a greater prevalence of RAE after this period. Furthermore, previous RAE studies in track and field showed a very low RAE or none at all in endurance disciplines for girls during and after the pubertal stage (Kearney et al., 2018; Brustio et al., 2019; Figueiredo et al., 2021), which could be due to the increase in oestrogen levels that decrease their relative endurance (Byrne and Hills, 2007; Manna, 2014).

Summarised, studies have documented that RAE exists within explosive track and field events. However, the prevalence of RAE in endurance events in track and field, such as hurdles and middle- and long-distance running, has scarcely been studied, especially in the youngest classes and among girls. Furthermore, few or no studies have been conducted of the RAE phenomenon in track and field that have specifically looked at the difference in the prevalence of RAE between the two physical characteristics of combined explosiveness and endurance. Another common feature of most RAE studies conducted on track and field is that they employ statistics that apply to a shorter period of time, which could potentially be a weakness as it could increase the likelihood that the results will be influenced by chance in cohorts. Given the aforementioned weaknesses, the purpose of the present study is to analyse the prevalence of RAE among the best Norwegian track and field athletes of all time in accordance with the two physical factors of explosiveness and endurance. More specifically, this study aims to investigate: (1) the extent to which RAE is present for the best Norwegian male and female track and field athletes of all time in the 13-year-old to senior (20 years and older) classes and (2) the extent to which a difference exists in RAE between explosive track and field events (sprint races, hurdles, high jump, pole vault, triple and long jump, discus and hammer throw, shot put and javelin) and endurance events in track and field (middle- and long-distance running and obstacle events).

## Materials and Methods

### Subjects

Included in this study are the top-100 Norwegian athletes of all time in each age class—i.e., from 13-years-old to seniors (20 years and older)—of both genders. The top 100 in all running, jumping and throwing events—apart from girls’ pole vault—are included (*N* = 21,711 across 221 independent samples). Of these, 11,384 are males and 10,327 are females. The sample was taken from the Norwegian Athletics Association’s website which has registers of the best athletes of all time (NFIF, 2018a^3^; NFIF, 2020^4^). The use of these data was approved in advance by the Norwegian Athletics Association. At the time of data collection, the statistics for the girls’ and boys’ classes (13–18 years) included data from the best performers up to 1 November 2018 (NFIF, 2018a^1^). For male seniors, all the events at the time of data collection were updated as of 1 January 2020 (NFIF, 2020b^2^), except for half marathons and marathons that were last updated on 1 January 2014 (NFIF, 2018a^1^). For female seniors, all running events, except half marathons and marathons that were updated on 1 January 2014, were updated as of 1 January 2020 (NFIF 2020^2^). The remainder of the events for female seniors were updated on 1 January 2014 (NFIF, 2018a^1^). The study was approved by the Norwegian Centre for Research Data, with reference number 217781.

### Procedure

First, the statistics of the Norwegian Athletics Association (NFIF, 2018a^1^, 2020^2^) were used to assign everyone’s month of birth to the entire sample (*N* = 21,711). The month of birth of the top 100 from both genders, in each age group and in all events, was recorded in Microsoft Excel and sorted by age class and event. In some track and field events, different rules have been in effect for conducting these events over the years, which means that the NFIF has several top-100 lists for each age class in some events. In cases where several top-100 statistics for an age group in an event exist, the rules that the different classes used in a championship context have been used (NFIF, 2020a^2^, 2021a^1^, b^3^). For example, in the long jump, it was common practice between 1979 and 1991 to measure jump length from a take-off zone, while before and after that period, the jump length has been measured from a take-off board. Therefore, separate top-100 statistics have been kept for both of these scenarios. In the shot put events, 3 kg was the usual weight for F13s (girls/female 13 years) during the 1970s and 1980s, while today 2 kg is the usual weight for this class of shot put events. In such cases, both the long jump and shot put, the top 50 from the top 100 with the old standard and the top 50 from the top 100 with the new standard have been collected to provide general figures for the best athletes of all time. If someone appeared on both lists (both old and new standards), they were only included from the top-100 list with the new standard since it was first registered and appeared first on the top-100 all-time list. Manual times for sprint events and those listed with “unapproved results” in events were omitted from the study. Fewer than 100 athletes were on the top-100 list for these events: 600 m for F13/F14/B14; 1,500 m for F15/F16; 2,000 m for F17; and 3,000 m for F18/F20, as these are fairly new events and only a few athletes had managed to achieve results that met the NFIF’s requirement for being included in the statistics. (See Appendix 1 for how the sample in the study is distributed, according to events and standards.)

### Statistics and Analysis

To investigate the presence of RAE among the best Norwegian track and field athletes of all time, the observed quarterly birth distribution was compared to an anticipated quarterly birth distribution. Microsoft Excel was used to register the birth month for each athlete and summarise how many athletes were born in each of the four quarters of the year (Q1: Jan-March; Q2: April–June; Q3: July-Sept; Q4: Oct-Dec). The anticipated quarterly distribution of births was taken from national data (SSB, 2020^5^). The number of quarterly live births in Norway during the period 1966–2019 was calculated using Statistics Norway’s public data (SSB, 2020) and used as the theoretically anticipated birth distribution: Q1: 25.00% (*n* = 785,175), Q2: 26.43% (*n* = 830,383), Q3: 25.62% (*n* = 804,908) and Q4: 22.95% (*n* = 720,789). To analyse the difference between observed and anticipated quarterly birth distribution chi-square tests (χ^2^) against an even distribution were used, with Cramer’s V (ϕ) as a measure of effect size (Statistics Kingdom, 2021^6^). The significance level was set at *p* < 0.05 and the effect sizes cut-off values were categorised at 0.1, 0.3 and 0.5 to be small, medium and large, respectively (Statistics Kingdom, 2020^8^).

## Results

### Subjects

**Figure 1:**
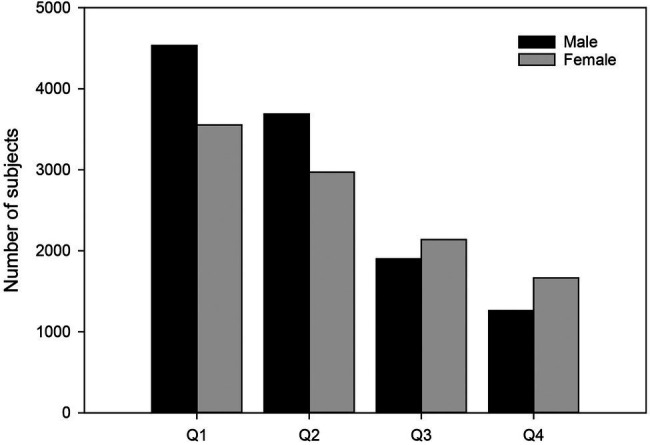
Quarterly birth distribution for males and females in the entire sample (*N* = 21,711). Q1 = Jan–March, Q2 = April–June, Q3 = July–Sept, Q4 = Oct–Dec. The number of subjects decreases significantly from Q1 to Q4 for both males *ɸ* = 0.44, *p* < 0.05, Large RAE and females *ɸ* = 0.26, Medium RAE, *p* < 0.05.

The prevalence of RAE among the best Norwegian track and field athletes of all ages in the 13-year-old to senior classes showed that the quarterly distribution of births in the entire sample (*N* = 21,711) was Q1 = 8,087, Q2 = 6,660, Q3 = 4,038 and Q4 = 2,926, which means that it deviated considerably from the quarterly birth distribution of the Norwegian population during the period 1966–2019 (*X*^2^ (3) = 2718.1, *ɸ* = 0.35, *p* < 0.05, medium RAE; see Figure 1).

### Age and Gender

RAE is strongest in the youngest examined class for both boys (*ɸ* = 0.69, *p* < 0.05) and girls (*ɸ* = 0.47, *p* < 0.05; Table 1). Furthermore, the results of the present study show that the RAE for boys is also strong in the 14-, 15-, 16- and 17-year-old classes (*ɸ* = 0.62–0.40), while for girls it is medium in the same classes (*ɸ* = 0.38–0.24). At the senior level, there is a small RAE in both men (*ɸ* = 0.18) and women (*ɸ* = 0.11; Table 1).

**Table 1:** Prevalence of RAE among the all time best Norwegian track and field athletes, by age and gender (*N* = 21,711).

Age category	Gender*	*n*	Q1	Q2	Q3	Q4	*X* ^2^	*P*	*ɸ*	RAE
13 years	M	1,400	711	435	173	81	659.6	<0.05	0.69	Large
	F	1,254	535	371	217	131	279.7	<0.05	0.47	Large
14 years	M	1,398	675	432	191	100	545.0	<0.05	0.62	Large
	F	1,264	491	374	229	170	179.2	<0.05	0.38	Medium
15 years	M	1,600	704	530	234	132	484.1	<0.05	0.55	Large
	F	1,469	528	414	305	222	125.4	<0.05	0.29	Medium
16 years	M	1,600	612	553	256	179	306.5	<0.05	0.44	Large
	F	1,467	520	403	316	228	108.7	<0.05	0.27	Medium
17 years	M	1700	639	564	303	194	276.3	<0.05	0.40	Large
	F	1,555	517	453	338	247	89.3	<0.05	0.24	Medium
18 years	M	1,686	601	558	296	231	210.8	<0.05	0.35	Medium
	F	1,513	454	434	321	304	35.1	<0.05	0.15	Small
Senior	M	2000	592	616	447	345	67.8	<0.05	0.18	Small
	F	1805	508	523	412	362	23.78	<0.05	0.11	Small
Total	M	11,384	4,534	3,688	1900	1,262	2206.7	<0.05	0.44	Large
	F	10,327	3,553	2,972	2,138	1,664	695.3	<0.05	0.26	Medium

* *M = males, F = females. Q*1 *= Jan–March, Q2 = April–June, Q3 = July–Sept, Q*4 *= Oct–Dec. *ɸ* = Cramers V. RAE = Relative age effect.*

### Degree of RAE in Explosive Events Versus Endurance Events

Of the total sample of 21,711, 5,600 (25.8%) came from throwing events, 4,875 (22.5%) from jumping events, 6,198 (28.5%) from sprint/hurdle events and 5,038 (23.2%) from medium- and long-distance events. This means that 76.8% of the sample came from explosive events, while the remaining 23.2% came from endurance events.

**Figure 2:**
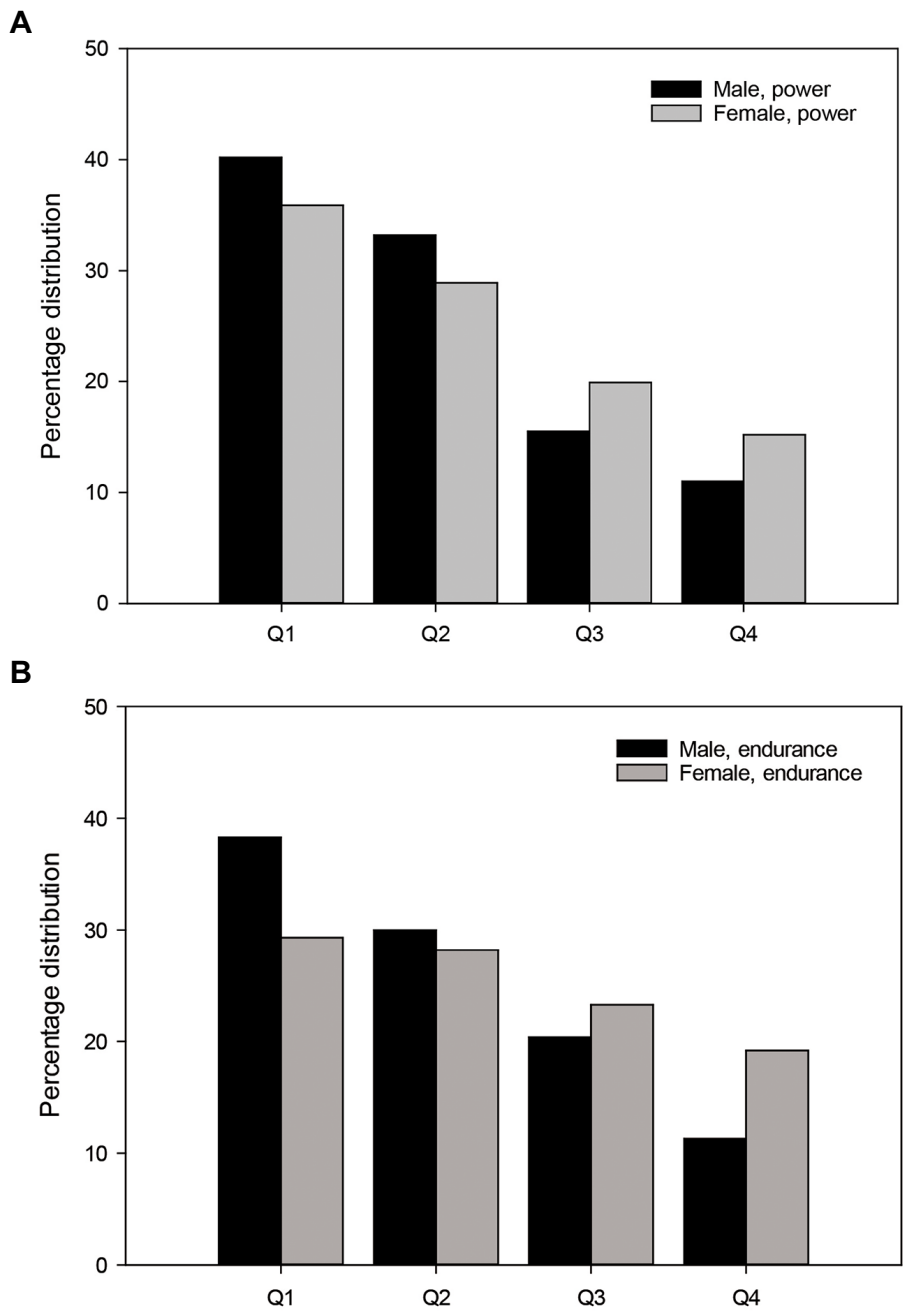
Percentage quarterly birth distribution for males and females in the power events **(A)** and endurance events **(B)**. Q1 = Jan-March, Q2 = April–June, Q3 = July–Sept, Q4 = Oct–Dec. Number of subjects decreases significantly from Q1 to Q4 for both males (*ɸ* = 0.46, *p* < 0.05, Large RAE and *ɸ* = 0.38, *p* < 0.05, Medium RAE) and females (*ɸ* = 0.30, Medium RAE, *p* < 0.05 and *ɸ* = 0.13, *p* < 0.05, Small RAE) in power and endurance events, respectively.

A high degree of RAE was found in explosive track and field events (Table 2; Figure 2A) There are large RAEs for both boys (*ɸ* = 0.67) and girls (*ɸ* = 0.49) in the 13-year-old class in explosive events, while in the 14, 15, 16 and 17-year-old classes there are still large RAEs for boys (*ɸ* = 0.60–0.43), but only medium RAEs for girls (*ɸ* = 0.40–0.28). Moreover, in the 18-year-old class (*ɸ* = 0.36) and the senior class (*ɸ* = 0.22), a medium RAE was found for males, while for females there was little RAE in both the 18-year-old class (*ɸ* = 0.16) and the senior class (*ɸ* = 0.11). In endurance track and field events, our results show that an RAE exists to a large extent for the males but not for the females (Figure 2B; Table 3). The 13-year-old class has a large RAE for the boys (*ɸ* = 0.81), whereas a medium RAE for the girls (*ɸ* = 0.37). In the other boys’ and girls’ classes, there is a large to medium RAE for the boys (*ɸ* = 0.76–0.34) and a medium to no RAE for the girls (*ɸ* = 0.25–0.12^*^). In the senior classes for the endurance events, there is little RAE for men (*ɸ* = 0.15) and no RAE for women (*ɸ* = 0.14^*^).

**Table 2:** Prevalence of RAE in the explosive events among the all time best Norwegian track and field athletes, by age and gender.

Age category	Gender*	*n*	Q1	Q2	Q3	Q4	*X* ^2^	*P*	*ɸ*	RAE
13 years	M	1,200	597	378	150	75	532.3	<0.05	0.67	Large
	F	1,100	474	328	186	112	259.2	<0.05	0.49	Large
14 years	M	1,200	564	381	163	92	435.3	<0.05	0.60	Large
	F	1,098	437	324	191	146	172.8	<0.05	0.40	Medium
15 years	M	1,200	539	398	163	100	390.7	<0.05	0.57	Large
	F	1,100	425	311	217	147	141.9	<0.05	0.36	Medium
16 years	M	1,200	451	431	180	138	238.4	<0.05	0.45	Large
	F	1,100	417	309	213	161	124.2	<0.05	0.34	Medium
17 years	M	1,300	494	447	211	148	238.9	<0.05	0.43	Large
	F	1,200	415	355	246	184	91.2	<0.05	0.28	Medium
18 years	M	1,300	462	437	222	179	167.4	<0.05	0.36	Medium
	F	1,200	366	343	252	239	31.5	<0.05	0.16	Small
Senior	M	1,300	394	416	262	228	61.3	<0.05	0.22	Medium
	F	1,175	329	338	284	224	15.5	0.0014	0.11	Small
Total	M	8,700	3,501	2,888	1,351	960	1842.3	<0.05	0.46	Large
	F	7,973	2,863	2,308	1,589	1,213	707.1	<0.05	0.30	Medium

* *M = males, F = females. Q*1 *= Jan–March, Q2 = April–June, Q3 = July–Sept, Q*4 *= Oct–Dec. *ɸ* = Cramers V. RAE = Relative age effect.*

**Table 3:** Prevalence of RAE in the endurance events among the all time best Norwegian track and field athletes, by age and gender.

Age category	Gender*	*n*	Q1	Q2	Q3	Q4	*X* ^2^	*P*	*ɸ*	RAE
13 years	M	200	114	57	23	6	132.4	<0.05	0.81	Large
	F	154	61	43	31	19	21.5	<0.05	0.37	Medium
14 years	M	198	111	51	28	8	115.2	<0.05	0.76	Large
	F	166	54	50	38	24	10.7	0.0135	0.25	Medium
15 years	M	400	165	132	71	32	97.2	<0.05	0.49	Large
	F	369	103	103	88	75	3.4	0.3369	0.09	None
16 years	M	400	161	122	76	41	74.5	<0.05	0.43	Large
	F	367	103	94	103	67	5.7	0.1266	0.12	None
17 years	M	400	141	122	90	47	42.6	<0.05	0.33	Medium
	F	355	102	98	92	63	6.1	0.1078	0.13	None
18 years	M	386	139	121	74	52	44.5	<0.05	0.34	Medium
	F	313	88	91	69	65	4.2	0.2361	0.12	None
Senior	M	700	198	200	185	117	16.5	0.0009	0.15	Small
	F	630	179	185	128	138	12.1	0.0071	0.14	Small
Total	M	2,684	1,029	805	547	303	391.9	<0.05	0.38	Medium
	F	2,354	690	664	549	451	39.7	<0.05	0.13	Small

* *M = boys/male, F = girls/female. Q*1 *= Jan-March, Q2 = April–June, Q3 = July-Sept, Q*4 *= Oct-Dec. *ɸ* = Cramers V. RAE = relative age effect.*

In summary, the RAE is strongest in explosive track and field events for both genders, with a stronger RAE for boys than for girls in both explosive and endurance events (Tables 2, 3). This is also evident when taking all the examined age classes in each gender into account, because then the RAE is large (*ɸ* = 0.46) for males in explosive events and medium (*ɸ* = 0.38) in endurance events, while for females the RAE (*ɸ* = 0.30) is medium in explosive events and small (*ɸ* = 0.13) in endurance events. See Appendixs 2A–C for the prevalence of the RAE in the running, throwing and jumping events, respectively.

## Discussion

The purpose of this study was to analyse the prevalence of RAE among the best Norwegian track and field athletes of all time aged 13 years to senior by age class, gender and physical characteristics of the events. The main results of the present study showed that the RAE is strongest in explosive events and in the youngest age groups for both genders. Furthermore, the results indicated that the RAE is also strong in endurance events for males of all classes except seniors, while for females there is a significant RAE only in the two youngest examined classes in endurance events.

This study found a larger RAE in boys than in girls in all the examined classes, a finding that was not fully supported by previous research. While the results of the present study show a large RAE for boys and a medium RAE for girls in the 16- and 17-year-classes, Redondo et al. (2019) analysed the grade of RAE among Spanish track and field athletes in the throwing disciplines found a large RAE for both boys and girls in the U18 class. Larger differences in the prevalence of RAE among genders in the present study can be due to the inclusion of endurance events, and previous research showed that being relatively older is not always beneficial for girls in endurance events (Kearney et al., 2018; Brustio et al., 2019). A plausible explanation for gender differences in RAE among the older adolescents classes is that the weight growth curve in boys and girls intersects at this age period, and girls overtake boys in weight (Malina and Bouchard, 1991). Consequently, girls will have an increase in relative adiposity (Malina et al., 2004a), which is a disadvantage in endurance sports since it weakens the relative endurance due to increased metabolic costs. Another finding in this study was that the greatest grade of RAE was found for the youngest examined class, the 13-year-old class, in both genders. The finding with the highest RAE in the youngest age groups is supported by several studies of RAE in sports, including the reviews by both Musch and Grondin (2001) and Smith et al. (2018), as well as studies that looked at the RAE phenomenon in track and field (Romann and Cobley, 2015; Kearney et al., 2018; Redondo et al., 2019). For example, Redondo et al. (2019), who examined the degree of RAE among Spanish elite throwers in athletics, found a large RAE in the U14 class in both boys and girls.

Several potential explanations can be postulated as to why the degree of RAE is high at the age of 13, and one of these is linked to physical maturation. Among 13-year-olds, for example, a potential 11-month age difference would account for 7% of life experience, meaning that those who are relatively older will have a good deal more movement experience than the relatively younger ones. Such a difference will mean a lot in athletics because performance in running, jumping and throwing events in track and field is primarily dependent on precisely the same basic skills—running, jumping and throwing—which comprise the play activities of children. Another plausible explanation as to why RAE is so heavily prevalent in the 13-year class, both in this study and that of Redondo et al. (2019), is that athletics is a popular sport both in Norway and in Spain. The popularity will therefore help to enable and explain a large RAE in these two studies, because competition between the athletes is becoming greater, which is supposed to promote increased RAE (Musch and Grondin, 2001; Wattie et al., 2015). For example, 13- and 14-year-old athletes in Norway compete every year to get selected to represent their region in the Lerøy-Games, which is the unofficial championship for 13- and 14-year-old track and field athletes in Norway.

Another interesting finding in the current study is that the RAE is small but also significant at the senior level for both men and women. The fact that the relative age effect is small at the senior level is that the differences resulting from physical maturation and puberty are no longer present (Musch and Grondin, 2001). Comparisons of the results of the current study with the results of Saavedra-García et al. (2016), who examined the degree of RAE in Spain’s best track and field athletes of all time, show a small degree of RAE in the senior class for both men and women in Spanish track and field athletes as well.

As regards the RAEs related to the different physical characteristics, explosiveness and endurance, this study found that the RAE is strongest in explosive events for both boys and girls and in endurance events the effect is strong for boys but not for girls. One finding of interest is that although the RAE is strongest in explosive events for males when looking at the RAE overall for all ages, the RAE is actually strongest in endurance events for boys aged 13 and 14. In the other examined age classes, the RAE is generally stronger in explosive events. Why the RAE is somewhat stronger in endurance events than in explosive events for boys aged 13 and 14 is not so easy to explain in a plausible manner; most likely the onset of puberty plays a significant role since this is the age when the boys reach puberty (Beunen and Malina, 2007). The effects of puberty will generate RAE in both explosive and endurance events because for boys it contributes to increased levels of the developmental hormone testosterone (Beunen and Malina, 2007; Handelsman, 2017). Testosterone improves strength and speed and also boosts endurance because increased testosterone levels lead to an increase in red blood cells (Bachman et al., 2010; Guo et al., 2013). However, puberty also makes people taller and heavier, which means that the conditions for performing well in athletic throwing events will improve (O’Connor et al., 2007), thus causing us to believe that RAE would be greater in explosive events than in endurance events. Nevertheless, the current results show that the RAE for boys is strongest in endurance events in the 13- and 14-year-old classes, which may be due to the fact that it takes a few years for the effects of puberty to be completed, and that its immediate effects are therefore equally beneficial in both explosive and endurance events.

This study also found that the RAE is stronger in explosive events than in endurance events for 15-, 16-, 17-18-year-old boys and for senior men. Brustio et al. (2019), who analysed the degree of RAE of the best track and field athletes in the world during the period 2007–2018, also found greater RAE in explosive track and field events such as the 100 m and triple jump than in endurance events such as the 1,500 m and 5,000 m in the U18 class for boys. There may be several reasons why the RAE becomes stronger in explosive events than in endurance events with increasing chronological age. The performance in explosive track and field events depend on more factors than do endurance events. For example, in throwing events, good technique will be important for performance in addition to the physical characteristics of explosiveness and strength (Carr, 1999; Zaras et al., 2019). Also, performance in endurance events, such as the 10,000 m run, primarily depend on endurance in terms of high maximum oxygen uptake (VO_2_ max), the lactate threshold and running economy (Bassett and Howley, 2000). The reason why it is conceivable that the RAE in this case is stronger in explosive events is that several studies have shown that relatively older athletes, in addition to having physical advantages, also have cognitive advantages compared to relatively younger competitors (Helsen et al., 2016), which means that it is possible that older athletes have had more time to learn appropriate techniques in explosive events. The reason why these advantages appear first in the 15-18-year-old classes and not the 13-14-year-old classes may be that it takes time to learn such additional skills. This conceivable explanation is supported by a review delving deeply into the RAE research conducted on boys in team sports (Cobley et al., 2009). This meta-analysis found the greatest RAE was at the ages of 15–18 in boys (OR *Q*1:*Q*4 = 2.36) and reasoned that performance in team sports depends on many skills, which take time to learn. Hence, our finding of greatest RAE in the ages 15–18 in boys in explosive events could be explained by the so-called adolescent awkwardness – phenomenon, which according to Lloyd et al. (2011) means that athletes get reduced motor coordination when they are close to Peak Height Velocity (PHV). Because 13- and 14 years – old athletes have a rapid growth of the limbs and trunks, they are close to PHV (Lloyd et al., 2011), and the normal advantage of being relatively older in explosive events would not necessary be significant since motor coordination is temporarily disrupted at this age. Furthermore, it is conceivable that those who are relatively older, who are taller, faster and stronger and who are often labelled as being talented, receive closer follow-up, feedback and expectations from their trainers, something which makes them even more likely to benefit from learning techniques in explosive events (Rosenthal and Jacobson, 1968). Consequently, this could result in Pygmalion and Galatea effects where the athletes are motivated to work harder to meet the expectations of their trainers, something which can contribute towards increased RAE.

When it comes to female athletes, the results show that the RAE is only large in the 13-year-old class in explosive events and that there is no significant RAE in the 15-, 16-, 17- and 18-year-old classes in endurance events. The results suggest that boys and girls are quite similar in development in terms of strength, speed and endurance up to puberty (Byrne and Hills, 2007; Malina et al., 2010; Manna, 2014). Average age of puberty start in Norwegian girls and boys are, respectively, 10.4 and 11.7 years (Oehme et al., 2020) and girls reach Peak Height Velocity (PHV) at the age of 11.5 years (Malina and Bouchard, 1991). High RAE in explosive events for girls in the 13-year-old class is therefore expected since they already have the puberty effect. However, it is a bit surprising that girls in the 13-year-old class have a medium RAE in endurance events. Especially since girls show an increase in endomorphic values, according to Malina et al., (2004a), which makes them less efficient in terms of cardiorespiratory response due to increased metabolic costs. Malina et al. (2010) examined the anthropometry and physical characteristics of track and field athletes aged 11–13 years and 14–15 years in both genders. They found that girls and boys 11–13 years old were on average equally tall and equally heavy, but boys performed somewhat better in the 20 m sprint, plyometrics and 2 kg ball events. Their findings may help to explain why the RAE is somewhat stronger in the 13-year class for boys in explosive track and field events in the present study.

In the 15-, 16-, 17- and 18-year-old classes for girls, the results of the study show a medium RAE in explosive events, but no RAE in endurance events. These findings are consistent with the findings of other research, such as the study conducted by Brustio et al. (2019), which found in the U18 class a small-to-medium RAE in explosive events such as the 100 m (OR = 1.8), hammer throw (OR = 2.5) and discus (OR = 2.6) but little or no RAE in endurance events such as the 800 m (OR = 1.4) and 5,000 m (OR = 1.5). The finding of a medium RAE in explosive events in the 15-, 16-, 17- and 18-year-old classes for girls is probably due to the fact that it is advantageous in throwing events to be relatively older, since puberty makes girls taller and heavier. Papaiakovou et al. (2009) found that girls grew 8 cm taller and became 5.5 kg heavier from the age of 12 to 13 and became even taller and heavier with increasing chronological age. This would be advantageous in throwing events since height and weight are important for generating power in the throwing motion (Carr, 1999; O’Connor et al., 2007). Relatively older girls often reach puberty earlier and will therefore become taller and heavier and obtain a physical head start on their relatively younger competitors in throwing events and therefore also gain advantages in the 14-, 15-, 16- and 17-year-old classes because puberty usually lasts for three to 4 years.

The fact that there is no RAE in endurance events for girls in the 15-, 16-, 17- and 18-year-old classes can be primarily explained by puberty combined with the physical work requirements of these disciplines. According to the requirements for distance running in track and field, VO_2_ max, measured in ml/kg/min, has a major significance for performance (Bassett and Howley, 2000; Midgley et al., 2007). For example, in high-level Spanish female long-distance runners, measurement of VO_2_ max was found to be 71.1 ml/kg/min and 72.9 ml/kg/min in 10,000 m and marathon runners, respectively (Legaz Arrese et al., 2005). The findings of Legaz Arrese and colleagues emphasise the importance of high relative endurance in endurance events for girls. Investigations have shown that being relatively older for girls limits their relative endurance (Baxter-Jones et al., 1993; Malina et al., 2010). A study conducted by Baxter-Jones et al. (1993) showed that before puberty female athletes have a VO_2_ max of 48.4 ml/kg/min, while those in the middle of puberty and at the end of puberty have a VO_2_ max of 48.2 and 44.7 ml/kg/min, respectively, a finding which suggests that relative endurance is impaired during puberty. Likewise, in orienteering, another sport in which relative endurance is important for performance, a study found no RAE (OR K1:K4 = 0.82) among high-level female orienteering runners aged 7–20 years (Romann et al., 2018). The effects of puberty and increasing fat mass on RAE in endurance events for girls was examined by Cobley et al. (2018) who studied the prevalence of RAE in girls for swimming, and for the 400 m freestyle endurance event, in particular. They found a small but significant RAE in 14-, 15-, 16- and 17-year-old girls and a medium RAE in J18. These results contradict the current study in which no RAE was found in track and field endurance events. This may be due to the fact that swimming and endurance events in track and field make demands on two different types of endurance. It is important when running to have good relative endurance since running is weight-bearing; this is not necessarily the case in swimming, which is not weight-bearing. A study conducted by Baxter-Jones et al. (1993), who studied the VO_2_ max of girls at different stages of puberty, found that l/min was 1.6 before puberty, 1.81 during puberty and 2.33 at the end of puberty, which supports the fact that endurance improves and that it can therefore be an advantage to be relatively older in endurance sports which are not weight-bearing, but not in running which features weight-bearing events.

### Strength and Limitations

The current study has some strengths that will contribute towards creating information of value in respect of RAE and research on talented athletes, including the fact that it focuses clearly on the distinction between the two physical characteristics of endurance and explosiveness. Yet another strength of the present study is that the samples are taken from an all-time best list, which affords the research a great methodical advantage because it enables all athletes that have been practicing athletics in Norway since the 1950s have had the opportunity to be on the list. Consequently, the results in this study will be very generalisable to the all-time best Norwegian track and field athletes. Furthermore, the use of all-time best lists will contribute to reducing the chances for coincidences in the different cohorts, which renders the results in the present study impervious of the charge of being influenced by particular cohorts. Despite its advantages, this study also has some gaps that future research should focus on filling. One weakness in this study is that 76.8% of those selected for the RAE analysis have been taken from explosive events, with only 23.3% taken from endurance events, something which could potentially affect comparison of the two physical factors. It may therefore be appropriate that further research on the relationship between RAE in track and field athletes has an equally large selection of athletes from the various events.

### Conclusion and Practical Applications

There is a high degree of RAE among the best Norwegian track and field athletes of all time in the youngest examined classes. With increasing chronological age, RAE subsides, but there is nevertheless small and significant RAE in the senior classes of both genders. Furthermore, RAE is more prevalent in explosive events than in endurance events in all classes for both genders, except for boys competing in the two youngest classes, 13 and 14 years. However, the results of the present study suggest that all track and field athletes, both those who are relatively older and those who are younger, should receive equal development opportunities when they are young, e.g., in the form of equal attention and equally close follow-up. While some athletes have better prerequisites than others, this is not necessarily seen in youth sports. In this way, it is highly likely that more people will choose to continue to be involved in track and field, and that those athletes who have great unresolved potential can be helped to realise their potential. Furthermore, the results indicate that selecting talented athletes and predicting who will be the best in adulthood based on their achievements at a young age is not expedient. Therefore, equal opportunities for development for everyone will be important and consequently it will be possible to prevent athletes with enormous potential from dropping out due to a lack of motivation to continue with sport in general and track and field in particular.

## Data Availability Statement

The original contributions presented in the study are included in the article/Supplementary Material, further inquiries can be directed to the corresponding author.

## Author Contributions

AK and TD: conception and idea of the study. AK: analysis and processing of data. AK, TD, and TR: writing the manuscript. TR, HG, and TD: critical supervision. All authors contributed to the article and approved the submitted version.

## Conflict of Interest

The authors declare that the research was conducted in the absence of any commercial or financial relationships that could be construed as a potential conflict of interest.

## Publisher’s Note

All claims expressed in this article are solely those of the authors and do not necessarily represent those of their affiliated organizations, or those of the publisher, the editors and the reviewers. Any product that may be evaluated in this article, or claim that may be made by its manufacturer, is not guaranteed or endorsed by the publisher.

## Footnotes

^1^ https://www.friidrett.no/aktivitet/nm-og-um/

^2^ https://www.friidrett.no/globalassets/barn-og-ungdom/ungdomslekene/leroy-lekene---konsept-og-reglement---versjon-aug2020.pdf

^3^ https://www.friidrett.no/aktivitet/statistikk/alle-tiders2/

^4^ http://www.minfriidrettsstatistikk.info/php/AlleTidersStatistikk.php

^5^ https://www.ssb.no/statbank/table/05531

^6^ https://www.statskingdom.com/310GoodnessChi.html
