# Supplementary material for: Relative Age Effect Among the Best Norwegian Track and Field Athletes of All Time: Comparisons of Explosive and Endurance Events
Source: Front Psychol. 2022 Jul 12;13:858095. doi: 10.3389/fpsyg.2022.858095 (PMC9315261; doi:10.3389/fpsyg.2022.858095)
Supplement: Supplementary file 1 [file Data_Sheet_1.docx]

Supplementary Material

**Appendix 1A**

*Illustrates how the samples from the running events are distributed, by class, gender, event and standard*

| Events | | J1J1F13 | F14 | F15 | F16 | F17 | F18 | W sr. | M13 | M14 | M15 | M16 | M17 | M18 | M sr. |  |  |  |  |  |  |  |
| --- | --- | --- | --- | --- | --- | --- | --- | --- | --- | --- | --- | --- | --- | --- | --- | --- | --- | --- | --- | --- | --- | --- |
|  | 60m | 100 | 100 |  |  |  |  |  | 100 | 100 |  |  |  |  |  |  |  |  |  |  |  |  |
|  | 100m |  |  | 100 | 100 | 100 | 100 | 100 |  |  | 100 | 100 | 100 | 100 | 100 |  |  |  |  |  |  |  |
|  | 200m | 100 | 100 | 100 | 100 | 100 | 100 | 100 | 100 | 100 | 100 | 100 | 100 | 100 | 100 |  |  |  |  |  |  |  |
|  | 400m |  |  |  |  | 100 | 100 | 100 |  |  |  |  | 100 | 100 | 100 |  |  |  |  |  |  |  |
|  | 600m | 54 | 66 |  |  |  |  |  | 100 | 98 |  |  |  |  |  |  |  |  |  |  |  |  |
|  | 800m |  |  | 100 | 100 | 100 | 100 | 100 |  |  | 100 | 100 | 100 | 100 | 100 |  |  |  |  |  |  |  |
|  | 1500m | 100 | 100 | 100 | 100 | 100 | 100 | 100 | 100 | 100 | 100 | 100 | 100 | 100 | 100 |  |  |  |  |  |  |  |
|  | 2000m |  |  | 100 |  |  |  |  |  |  | 100 |  |  |  |  |  |  |  |  |  |  |  |
|  | 3000m |  |  |  | 100 | 100 | 100 |  |  |  |  | 100 | 100 |  |  |  |  |  |  |  |  |  |
|  | 5000m |  |  |  |  |  |  | 100 |  |  |  |  |  | 100 | 100 |  |  |  |  |  |  |  |
|  | 10000m |  |  |  |  |  |  | 100 |  |  |  |  |  |  | 100 |  |  |  |  |  |  |  |
|  | Half marathon |  |  |  |  |  |  | 100 |  |  |  |  |  |  | 100 |  |  |  |  |  |  |  |
|  | Marathon |  |  |  |  |  |  | 100 |  |  |  |  |  |  | 100 |  |  |  |  |  |  |  |
|  | 1500mh |  |  | 69 | 67 |  |  |  |  |  | 100 | 100 |  |  |  |  |  |  |  |  |  |  |
|  | 2000mh |  |  |  |  | 55 |  |  |  |  |  |  | 100 |  |  |  |  |  |  |  |  |  |
|  | 3000mh |  |  |  |  |  | 13 | 30 |  |  |  |  |  | 86 | 100 |  |  |  |  |  |  |  |
|  | 60mh 76.2cm | 100 | 100 |  |  |  |  |  | 100 |  |  |  |  |  |  |  |  |  |  |  |  |  |
|  | 80mh 76.2cm |  |  | 100 | 100 |  |  |  |  | 50 |  |  |  |  |  |  |  |  |  |  |  |  |
|  | 80mh 84cm |  |  |  |  |  |  |  |  | 50 |  |  |  |  |  |  |  |  |  |  |  |  |
|  | 100mh 76.2 cm |  |  |  |  | 100 |  |  |  |  |  |  |  |  |  |  |  |  |  |  |  |  |
|  | 100mh 84cm |  |  |  |  |  | 100 | 100 |  |  | 28 |  |  |  |  |  |  |  |  |  |  |  |
|  | 100mh 91.4cm |  |  |  |  |  |  |  |  |  | 72 | 100 | 50 |  |  |  |  |  |  |  |  |  |
|  | 110mh 100cm |  |  |  |  |  |  |  |  |  |  |  | 50 | 100 |  |  |  |  |  |  |  |  |
|  | 110mh 106.7cm |  |  |  |  |  |  |  |  |  |  |  |  |  | 100 |  |  |  |  |  |  |  |
|  | 200mh 68cm | 100 |  |  |  |  |  |  | 100 |  |  |  |  |  |  |  |  |  |  |  |  |  |
|  | 200mh 76.2cm |  | 98 |  |  |  |  |  |  | 100 |  |  |  |  |  |  |  |  |  |  |  |  |
|  | 300mh 76.2cm |  |  | 100 | 100 | 100 |  |  |  |  | 50 | 100 |  |  |  |  |  |  |  |  |  |  |
|  | 300mh 84 cm |  |  |  |  |  |  |  |  |  | 50 |  | 50 |  |  |  |  |  |  |  |  |  |
|  | 300mh 91.4 cm |  |  |  |  |  |  |  |  |  |  |  | 50 |  |  |  |  |  |  |  |  |  |
|  | 400mh 76.2 cm |  |  |  |  |  | 100 | 100 |  |  |  |  |  |  |  |  |  |  |  |  |  |  |
|  | 400mh 91.4 cm |  |  |  |  |  |  |  |  |  |  |  |  | 100 | 100 |  |  |  |  |  |  |  |
|  | *Note. * M = males, F = females* | | | | | | | | | | | | | | | |  |  |  |  |  |  |

**Appendix 1B**

*Illustrates how the samples from the jumping and throwing events are distributed, by class, gender, event and standard*

| Events | J1J1F13 | | F14 | | F15 | | F16 | | F17 | | F18 | | W sr. | | GM13 | M14 | | M15 | | M16 | | M17 | | M18 | M sr. | |  | |  | |  | |  | |  | |  | |  |
| --- | --- | --- | --- | --- | --- | --- | --- | --- | --- | --- | --- | --- | --- | --- | --- | --- | --- | --- | --- | --- | --- | --- | --- | --- | --- | --- | --- | --- | --- | --- | --- | --- | --- | --- | --- | --- | --- | --- | --- |
| Long jump | 50 | | | 50 | | 100 | | 100 | | 100 | | 100 | | 100 | 50 | | 50 | | 100 | | 100 | | 100 | 100 | | 100 |  |  | |  | |  | |  | |  | |  | |
| Long jump old | 50 | | | 50 | |  | |  | |  | |  | |  | 50 | | 50 | |  | |  | |  |  | |  |  |  | |  | |  | |  | |  | |  | |
| High jump | 100 | | | 100 | | 100 | | 100 | | 100 | | 100 | | 75 | 100 | | 100 | | 100 | | 100 | | 100 | 100 | | 100 |  |  | |  | |  | |  | |  | |  | |
| Pole Vault |  | | |  | |  | |  | |  | |  | |  | 100 | | 100 | | 100 | | 100 | | 100 | 100 | | 100 |  |  | |  | |  | |  | |  | |  | |
| Triple jump | 50 | | | 50 | | 100 | | 100 | | 100 | | 100 | | 100 | 50 | | 50 | | 100 | | 100 | | 100 | 100 | | 100 |  |  | |  | |  | |  | |  | |  | |
| Triple jump old | 50 | | | 50 | |  | |  | |  | |  | |  | 50 | | 50 | |  | |  | |  |  | |  |  |  | |  | |  | |  | |  | |  | |
| Discus throw 0.6kg | 50 | | |  | |  | |  | |  | |  | |  |  | |  | |  | |  | |  |  | |  |  |  | |  | |  | |  | |  | |  | |
| Discus throw 0.75kg |  | | | 50 | | | |  | |  | |  | |  | 44 | |  | |  | |  | |  |  | |  |  |  | |  | |  | |  | |  | |  | |
| Discus throw 1kg | 50 | | | 50 50 | | 100 | | 100 | | 1100 | | 100 | | 100 | 56 | | 100 | | 50 | |  | |  |  | |  |  |  | |  | |  | |  | |  | |  | |
| Discus throw 1,5kg |  |  | |  | |  | |  | |  | |  | |  |  | |  | | 50 | | 100 | | 100 |  | |  |  |  | |  | |  | |  | |  | |  | |
| Discus throw 1.75kg |  |  | |  | |  | |  | |  | |  | |  |  | |  | |  | |  | |  | 50 | |  |  |  | |  | |  | |  | |  | |  | |
| Discus throw 2kg |  |  | |  | |  | |  | |  | |  | |  |  | |  | |  | |  | |  | 50 | | 100 |  |  | |  | |  | |  | |  | |  | |
| Hammer 3kg/110 | 100 | | | 50 | |  | |  | |  | |  | |  | 50 | |  | |  | |  | |  |  | |  |  |  | |  | |  | |  | |  | |  | |
| Hammer 3kg/119.5 |  |  | | 50 | |  | |  | |  | |  | |  |  | |  | |  | |  | |  |  | |  |  |  | |  | |  | |  | |  | |  | |
| Hammer 4kg/110 |  |  | |  | | 100 | | 100 | | 100 | |  | |  | 50 | | 50 | | 50 | |  | |  |  | |  |  |  | |  | |  | |  | |  | |  | |
| Hammer 4kg/119.5 |  |  | |  | |  | |  | |  | | 100 | | 100 |  | | 50 | | 50 | |  | |  |  | |  |  |  | |  | |  | |  | |  | |  | |
| Hammer 6.25kg |  |  | |  | |  | |  | |  | |  | |  |  | |  | |  | | 100 | | 100 | 100 | |  |  |  | |  | |  | |  | |  | |  | |
| Hammer 7.26kg |  |  | |  | |  | |  | |  | |  | |  |  | |  | |  | |  | |  |  | | 100 |  |  | |  | |  | |  | |  | |  | |
| Shot put 2kg | 50 | | |  | |  | |  | |  | |  | |  |  | |  | |  | |  | |  |  | |  |  |  | |  | |  | |  | |  | |  | |
| Shot put 3kg | 50 | | | 100 | | 100 | | 100 | | 50 | |  | |  | 100 | |  | |  | |  | |  |  | |  |  |  | |  | |  | |  | |  | |  | |
| Shot put 4kg |  |  | |  | |  | |  | | 50 | | 100 | | 100 |  | | 100 | | 100 | |  | |  |  | |  |  |  | |  | |  | |  | |  | |  | |
| Shot put 5kg |  |  | |  | |  | |  | |  | |  | |  |  | |  | |  | | 50 | | 46 |  | |  |  |  | |  | |  | |  | |  | |  | |
| Shot put 5.5kg |  |  | |  | |  | |  | |  | |  | |  |  | |  | |  | | 50 | | 54 |  | |  |  |  | |  | |  | |  | |  | |  | |
| Shot put 6kg |  |  | |  | |  | |  | |  | |  | |  |  | |  | |  | |  | |  | 43 | |  |  |  | |  | |  | |  | |  | |  | |
| Shot put.2kg |  |  | |  | |  | |  | |  | |  | |  |  | |  | |  | |  | |  | 57 | | 100 |  |  | |  | |  | |  | |  | |  | |
| Javelin 400g | 100 | | | 100 | |  | |  | |  | |  | |  | 50 | |  | |  | |  | |  |  | |  |  |  | |  | |  | |  | |  | |  | |
| Javelin 500g |  |  | |  | | 50 | |  | |  | |  | |  |  | |  | |  | |  | |  |  | |  |  |  | |  | |  | |  | |  | |  | |
| Javelin 600g |  |  | |  | | 50 | | 100 | | 100 | | 100 | | 50 | 50 | | 100 | | 100 | | 50 | |  |  | |  |  |  | |  | |  | |  | |  | |  | |
| Javelin 600g old |  |  | |  | |  | |  | |  | |  | | 50 |  | |  | |  | |  | |  |  | |  |  |  | |  | |  | |  | |  | |  | |
| Javelin 700g |  |  | |  | |  | |  | |  | |  | |  |  | |  | |  | | 50 | |  |  | |  |  |  | |  | |  | |  | |  | |  | |
| Javelin 800g |  |  | |  | |  | |  | |  | |  | |  |  | |  | |  | |  | | 50 | 50 | | 50 |  |  | |  | |  | |  | |  | |  | |
| Javelin 800g old |  |  | |  | |  | |  | |  | |  | |  |  | |  | |  | |  | | 50 | 50 | | 50 |  |  | |  | |  | |  | |  | |  | |
| *Note. * M = males, F = females* | | | | | | | | | | | | | | | | | | | | |  | |  |  | |  |  |  | |  | |  | |  | |  | |  | |

| Appendix 2A | | | | | | | | | | | | | | |
| --- | --- | --- | --- | --- | --- | --- | --- | --- | --- | --- | --- | --- | --- | --- |
| *Prevalence of RAE in the running events, by age category and gender among the all time best Norwegian track and field athletes* | | | | | | | | | | | |  | |  |
| Age category | | Event | Gender* | n | Q1 | Q2 | Q3 | Q4 | X^2^ | P | *ɸ* | | RAE | |
|  | | Sprint | M | 400 | 206 | 107 | 58 | 29 | 174,5 | <0,05 | 0.66 | | Large | |
|  |  |  | F | 400 | 176 | 120 | 70 | 34 | 106,2 | <0.05 | 0.52 | | Large | |
| 13 years | | Middle | M | 100 | 61 | 29 | 8 | 2 | 83,8 | <0,05 | 0,92 | | Large | |
|  |  |  | F | 54 | 25 | 11 | 13 | 5 | 13,4 | 0,0037 | 0,50 | | Large | |
|  | | Long | M | 100 | 53 | 28 | 15 | 4 | 51,9 | <0,05 | 0,72 | | Large | |
|  |  |  | F | 100 | 36 | 32 | 18 | 14 | 12,2 | 0,0067 | 0,35 | | Medium | |
|  | | Sprint | M | 400 | 195 | 115 | 63 | 27 | 151,8 | <0,05 | 0,62 | | Large | |
|  |  |  | F | 398 | 142 | 132 | 70 | 54 | 49,7 | <0,05 | 0,35 | | Medium | |
| 14 years | | Middle | M | 98 | 59 | 23 | 12 | 4 | 68,1 | <0,05 | 0,83 | | Large | |
|  |  |  | F | 66 | 25 | 12 | 19 | 10 | 7,1 | 0,0676 | 0,33 | | None | |
|  | | Long | M | 100 | 52 | 28 | 16 | 4 | 48,9 | <0,05 | 0,70 | | Large | |
|  |  |  | F | 100 | 29 | 38 | 19 | 14 | 11,6 | 0,0089 | 0,34 | | Medium | |
|  |  | Sprint | M  F | 400 | 179 | 116 | 74 | 31 | 111,5 | <0,05 | 0,53 | | Large | |
|  |  |  |  | 400 | 148 | 123 | 80 | 49 | 50,6 | <0,05 | 0,36 | | Medium | |
| 15 years | | Middle | M  F | 200 | 88 | 69 | 31 | 12 | 66,7 | <0,05 | 0,58 | | Large | |
|  |  |  |  | 200 | 58 | 54 | 48 | 40 | 2,3 | 0,5206 | 0,11 | | None | |
|  | | Long | M  F | 200 | 77 | 63 | 40 | 20 | 33,5 | <0,05 | 0,41 | | Large | |
|  |  |  |  | 169 | 45 | 49 | 40 | 35 | 1,2 | 0,7555 | 0,084 | | None | |
|  | | Sprint | M  F | 400 | 156 | 127 | 67 | 50 | 66,7 | <0,05 | 0,41 | | Large | |
|  |  |  |  | 400 | 134 | 132 | 78 | 56 | 37,7 | <0,05 | 0,31 | | Medium | |
| 16 years | | Middle | M  F | 200 | 92 | 58 | 34 | 16 | 61,0 | <0,05 | 0,55 | | Large | |
|  |  |  |  | 200 | 58 | 47 | 59 | 36 | 5,4 | 0,1455 | 0,16 | | None | |
|  | | Long | M  F | 200 | 69 | 64 | 42 | 25 | 20,7 | 0,0001 | 0,32 | | Medium | |
|  |  |  |  | 167 | 45 | 47 | 44 | 31 | 1,7 | 0,6299 | 0,10 | | None | |
|  | | | | | | | | | | | | | | |
|  | | Sprint | M | 500 | 194 | 160 | 93 | 53 | 87,0 | <0,05 | 0,42 | | Large | |
|  |  |  | F | 500 | 159 | 160 | 107 | 74 | 33,3 | <0,05 | 0,26 | | Medium | |
| 17 years | | Middle | M | 200 | 75 | 58 | 46 | 21 | 27,0 | 0,000 | 0,37 | | Medium | |
|  |  |  | F | 200 | 57 | 51 | 55 | 37 | 3,1 | 0,372 | 0,13 | | None | |
|  | | Long | M | 200 | 66 | 64 | 44 | 26 | 17,1 | 0,0006 | 0,29 | | Medium | |
|  |  |  | F | 155 | 45 | 47 | 37 | 26 | 4,3 | 0,227 | 0,17 | | None | |
|  | | Sprint | M | 500 | 189 | 152 | 95 | 64 | 66,9 | <0,05 | 0,37 | | Medium | |
|  |  |  | F | 500 | 132 | 153 | 100 | 115 | 9,9 | 0,0198 | 0,14 | | Small | |
| 18 years | | Middle | M | 200 | 77 | 59 | 40 | 24 | 28,2 | 0,000 | 0,38 | | Medium | |
|  |  |  | F | 200 | 55 | 55 | 49 | 41 | 1,2 | 0,7536 | 0,077 | | None | |
|  | | Long | M | 186 | 62 | 62 | 34 | 28 | 18,3 | 0,0003 | 0,31 | | Medium | |
|  |  |  | F | 113 | 33 | 36 | 20 | 24 | 5,0 | 0,1689 | 0,21 | | None | |
|  |  | Sprint | M  F | 500 | 144 | 155 | 110 | 91 | 14,4 | 0,0023 | 0,17 | | Small | |
|  |  |  |  | 500 | 129 | 140 | 134 | 97 | 3,7 | 0,2944 | 0,086 | | None | |
| Senior | | Middle | M  F | 200 | 53 | 60 | 51 | 36 | 3,3 | 0,3507 | 0,13 | | None | |
|  |  |  |  | 200 | 59 | 60 | 33 | 48 | 9,0 | 0,0295 | 0,21 | | Medium | |
| Long | M  F | 500 | 145 | 140 | 134 | 81 | 14,0 | 0,0028 | 0,17 | | Small | |  |  |
|  |  | 430 | 120 | 125 | 95 | 90 | 5,5 | 0,1384 | 0,11 | | None | |  |  |
| Sprint | M  F | 3100 | 1 263 | 932 | 560 | 345 | 579,9 | <0,05 | 0,43 | | Large | |  |  |
|  |  | 3098 | 1 020 | 960 | 639 | 479 | 208,4 | <0,05 | 0,26 | | Medium | |  |  |
| Total | | Middle | M  F | 1198 | 505 | 356 | 222 | 115 | 263,3 | <0,05 | 0,47 | | Large | |
|  |  |  |  | 1120 | 337 | 290 | 276 | 217 | 18,4 | 0,0003 | 0,13 | | Small | |
|  | | Long | M  F | 1486 | 524 | 449 | 325 | 188 | 148,0 | <0,05 | 0,32 | | Medium | |
|  |  |  |  | 1234 | 353 | 374 | 273 | 234 | 27,7 | <0,05 | 0,15 | | Small | |
| *Note. **M = males, F = females. Q1 = Jan-March, Q2 = April-June, Q3 = July-Sept, Q4 = Oct-Dec. *ɸ* = Cramers V. RAE = relative age effect | | | | | | | | | | | | | | |

| Appendix 2B | | | | | | | | | | | | | |
| --- | --- | --- | --- | --- | --- | --- | --- | --- | --- | --- | --- | --- | --- |
| *Prevalence of RAE in the throwing events, by age category and gender among the all time best Norwegian track and field athletes* | | | | | | | | | | |  | |  |
| Age category |  | Gender* | n | Q1 | Q2 | Q3 | Q4 | X^2^ | P | *ɸ* | | RAE | |
| 13 years |  | M | 400 | 196 | 130 | 50 | 24 | 174.4 | <0.05 | 0.66 | | Large | |
|  |  | F | 400 | 158 | 115 | 74 | 53 | 58.6 | <0.05 | 0.38 | | Medium | |
| 14 years |  | M | 400 | 178 | 145 | 53 | 24 | 149.0 | <0.05 | 0.61 | | Large | |
|  |  | F | 400 | 173 | 98 | 67 | 62 | 75.7 | <0.05 | 0.43 | | Large | |
| 15 years |  | M | 400 | 173 | 152 | 45 | 30 | 146.9 | <0.05 | 0.61 | | Large | |
|  |  | F | 400 | 155 | 108 | 79 | 58 | 48.0 | <0.05 | 0.35 | | Medium | |
| 16 years |  | M | 400 | 138 | 166 | 54 | 42 | 98.2 | <0.05 | 0.50 | | Large | |
|  |  | F | 400 | 159 | 101 | 77 | 63 | 50.3 | <0.05 | 0.35 | | Medium | |
| 17 years |  | M | 400 | 145 | 157 | 64 | 34 | 95.5 | <0.05 | 0.49 | | Large | |
|  |  | F | 400 | 149 | 111 | 78 | 62 | 39.7 | <0.05 | 0.31 | | Medium | |
| 18 years |  | M | 400 | 139 | 157 | 61 | 43 | 82.3 | <0.05 | 0.45 | | Large | |
|  |  | F | 400 | 138 | 111 | 85 | 66 | 24.9 | <0.05 | 0.25 | | Medium | |
| Senior |  | M  F | 400 | 136 | 138 | 64 | 62 | 46.6 | <0.05 | 0.34 | | Medium | |
|  |  |  | 400 | 123 | 114 | 87 | 76 | 10.9 | 0.0123 | 0.16 | | Small | |
| Total |  | M  F | 2 800 | 1 105 | 1 045 | 391 | 259 | 737.6 | <0.05 | 0.51 | | Large | |
|  |  |  | 2 800 | 1 055 | 758 | 547 | 440 | 284.9 | <0.05 | 0.32 | | Medium | |
| *Note. **M = males, F = females. Q1 = Jan-March, Q2 = April-June, Q3 = July-Sept, Q4 = Oct-Dec.  *ɸ* = Cramers V. RAE = relative age effect | | | | | | | | | | | | | |

| Appendix 2C | | | | | | | | | | | | | |
| --- | --- | --- | --- | --- | --- | --- | --- | --- | --- | --- | --- | --- | --- |
| *Prevalence of RAE in the jumping events, by age category and gender among the all time best Norwegian track and field athletes* | | | | | | | | | | |  | |  |
| Age category |  | Gender* | n | Q1 | Q2 | Q3 | Q4 | X^2^ | P | *ɸ* | | RAE | |
| 13 years |  | M | 400 | 195 | 141 | 42 | 22 | 190.4 | <0.05 | 0.69 | | Large | |
|  |  | F | 300 | 140 | 93 | 42 | 25 | 102.8 | <0.05 | 0.59 | | Large | |
| 14 years |  | M | 400 | 191 | 121 | 47 | 41 | 142.9 | <0.05 | 0.60 | | Large | |
|  |  | F | 300 | 122 | 94 | 54 | 30 | 61.2 | <0.05 | 0.45 | | Large | |
| 15 years |  | M | 400 | 187 | 130 | 44 | 39 | 144.6 | <0.05 | 0.60 | | Large | |
|  |  | F | 300 | 122 | 80 | 58 | 40 | 46.3 | <0.05 | 0.39 | | Medium | |
| 16 years |  | M | 400 | 157 | 138 | 59 | 46 | 83.3 | <0.05 | 0.46 | | Large | |
|  |  | F | 300 | 124 | 76 | 58 | 42 | 47.4 | <0.05 | 0.40 | | Medium | |
| 17 years |  | M | 400 | 159 | 125 | 56 | 60 | 70.1 | <0.05 | 0.42 | | Large | |
|  |  | F | 300 | 107 | 84 | 61 | 48 | 23.7 | <0.05 | 0.28 | | Medium | |
| 18 years |  | M | 400 | 134 | 128 | 66 | 72 | 33.2 | <0.05 | 0.29 | | Medium | |
|  |  | F | 300 | 96 | 79 | 67 | 58 | 8.9 | <0.05 | 0.17 | | Small | |
| Senior |  | M  F | 400 | 114 | 123 | 88 | 75 | 9.7 | 0.020 | 0.16 | | Small | |
|  |  |  | 275 | 77 | 84 | 63 | 51 | 5.6 | 0.1345 | 0.14 | | None | |
| Total |  | M  F | 2800 | 1137 | 906 | 402 | 355 | 577.4 | <0.05 | 0.45 | | Large | |
|  |  |  | 2075 | 788 | 590 | 403 | 294 | 243.5 | <0.05 | 0.34 | | Medium | |
| *Note. **M = males, F = females. Q1 = Jan-March, Q2 = April-June, Q3 = July-Sept, Q4 = Oct-Dec.  *ɸ* = Cramers V. RAE = relative age effect | | | | | | | | | | | | | |
